# Emphysema-Predominant COPD Had a Greater 5-Year Mortality and a Worse Annual Decline in Lung Function Than Airway Obstruction-Predominant COPD or Asthma at Initial Same Degree of Airflow Obstruction

**DOI:** 10.3390/medicina57111261

**Published:** 2021-11-17

**Authors:** Chang-Wei Lin, Hung-Yu Huang, Fu-Tsai Chung, Chun-Yu Lo, Yu-Chen Huang, Ting-Wen Wang, Lan-Yan Yang, Yu-Bin Pan, Kian Fan Chung, Chun-Hua Wang

**Affiliations:** 1 Department of Thoracic Medicine, Chang Gung Memorial Hospital, Taipei 105, Taiwan; naturewei@cgmh.org.tw (C.-W.L.); compaction71@gmail.com (H.-Y.H.); vikingchung@yahoo.com.tw (F.-T.C.); mixova@yahoo.com (C.-Y.L.); yuchenhahaha@cgmh.org.tw (Y.-C.H.); 2 College of Medicine, Chang Gung University, Taoyuan 333, Taiwan; 3 Department of Physical Medicine and Rehabilitation, National Taiwan University Hospital, Taipei 100, Taiwan; rr224456jp@gmail.com; 4 Biostatistics Unit, Clinical Trial Center, Chang Gung Memorial Hospital, Taoyuan 333, Taiwan; lyyang0111@gmail.com (L.-Y.Y.); e8901145@gmail.com (Y.-B.P.); 5 National Heart & Lung Institute, Imperial College London & Royal Brompton Hospital, London SW3 6LY, UK; f.chung@imperial.ac.uk

**Keywords:** chronic obstructive pulmonary disease, asthma, emphysema, 6 min walk test, pulmonary function, desaturation, mortality

## Abstract

*Background and Objectives:* We studied whether the extent of exertional oxygen desaturation and emphysema could cause greater mortality in COPD and asthma independent of airflow obstruction. *Materials and Methods:* We performed a 5-year longitudinal observational study in COPD and asthma patients who matched for airflow obstruction severity. All subjects performed a 6-min walk test (6MWT) and high-resolution computed tomography (HRCT) and followed spirometry and oxygen saturation (SpO_2_) during the 6MWT every 3–6 months. Overall survival was recorded. Cumulative survival curves were performed according to the Kaplan–Meier method and compared with the log-rank test. *Results:* The COPD group had higher emphysema scores, higher Δinspiratory capacities (ICs) and lower SpO_2_ during the 6MWT, which showed a greater yearly decline in FEV_1_ (40.6 mL) and forced vital capacity (FVC) (28 mL) than the asthma group (FEV_1_, 9.6 mL; FVC, 1.2 mL; *p* < 0.05). The emphysema-predominant COPD group had an accelerated annual decline in lung function and worse survival. The nadir SpO_2_ ≤ 80% and a higher emphysema score were the strong risk factors for mortality in COPD patients. *Conclusions:* The greater structural changes with a higher emphysema score and greater desaturation during the 6MWT in COPD may contribute to worse yearly decline in FEV_1_ and higher five-year mortality than in asthma patients with a similar airflow obstruction. The lowest SpO_2_ ≤ 80% during the 6MWT and emphysema-predominant COPD were the strong independent factors for mortality in chronic obstructive airway disease patients.

## 1. Introduction

Chronic obstructive pulmonary disease (COPD) and asthma are two of the most common chronic obstructive airway diseases and are characterized by inflammatory airway disease and airflow limitation [1], but with differences in immunology and physiology [1,2]. The distinct patterns of airway inflammation and structural changes underlying the pathology of asthma and COPD could contribute to airway resistance or alveolar loss and the loss of lung elastic recoil [3,4], thus leading to different clinical outcomes, even at the same airflow limitation. In COPD, this is accompanied by a reduction in alveolar attachments on airways or pulmonary gas exchange abnormalities, resulting in exercise-induced desaturation [5,6]. Some patients with persistent obstructive asthma may develop an irreversible airflow limitation [7,8], similar to the airflow limitation found in COPD. However, the airflow limitation in COPD is poorly reversed by currently available bronchodilators and is associated with an accelerated decline in lung function over and above the normal aging process [9].

Increased mortality in asthma patients is associated with the male gender, current smoking status, a lower forced expiratory volume in one second (FEV_1_) and a large degree of β-agonist-induced bronchodilation in population-based studies [10,11,12]. Risk factors, including lower FEV_1_, age, body mass index (BMI), dyspnea, reduced exercise capacity, frequent acute exacerbations and a high BODE index, have also been reported to be associated with mortality in COPD patients [13,14,15]. Within another study, the presence of emphysema and airway disease, as assessed using computed tomography and decreased pulmonary function, were particularly high-risk factors associated with death in COPD patients [16]. The 6-minute walk test (6MWT) is frequently used to assess the functional performance of patients with COPD in terms of walking distance and oxygen desaturation [17], and oxygen desaturation during the 6MWT has also been shown to be a predictor of mortality [18,19]. We have shown that dynamic hyperinflation in COPD decreased the levels of exhaled nitric oxide after the 6MWT, while the changes in exhaled NO in asthma after the 6MWT were highly associated with the severity of small airway obstruction; this may differentiate the underlying airway obstruction of asthma or COPD patients, which may be a factor for distinctive patterns of airway inflammation and structural changes from the two diseases [20]. However, patients with COPD or severe asthma overlap syndrome have a higher mortality compared to those with asthma alone [8,21], and the presence of emphysema is a poor prognostic factor for COPD patients [13,14,15,16,22,23,24]. The aim of the study was to evaluate whether the desaturation during the 6MWT and COPD with predominant emphysema would be a risk factor for mortality in COPD and chronic asthma patients at initial similar airflow obstruction and age match.

## 2. Material and Methods

### 2.1. Study Population

**Figure 1:**
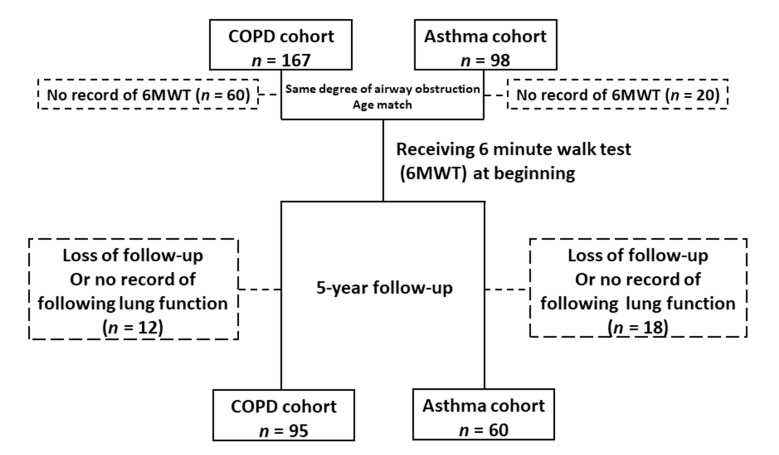
Study flow chart. Abbreviations: COPD, chronic obstructive pulmonary disease.

This study retrospectively evaluated clinical records of 167 patients with COPD and 98 with asthma at the outpatient clinic of Chang Gung Memorial Hospital between 2008 and 2011. The study flow is displayed in Figure 1. Asthma was ascertained from a clinical history of reversible airway obstruction with an increase in forced expiratory volume in one second (FEV_1_) of ≥12% or more than 200 mL [25] and regularly using anti-asthma treatment. The diagnosis of COPD was confirmed using spirometry, with a post-bronchodilator FEV_1_/forced vital capacity (FVC) ratio of less than 70% in the absence of a significant rise in FEV_1_ (12% and 200 mL) after inhalation of bronchodilators, according to GOLD guidelines [9], and those aged less than 40 were excluded. Patients who were diagnosed with bronchiectasis, cystic fibrosis, upper airways obstruction, bronchiolitis related to systemic disease or severe systemic diseases, such as hematologic disease, malignancy, systemic lupus erythema, end stage of renal disease and severe liver cirrhosis, were excluded. To avoid selection bias, all COPD and asthma patients had a similar severity of FEV_1_ and age match at entry. After a review of their clinical medical records, 60 COPD and 20 asthma subjects who had no record of a 6-minute walk test (6MWT) were excluded. Each subject of the cohort who had performed a 6MWT at the beginning was enrolled. Then, those patients with airway diseases would perform either a 6MWT or spirometry every 3 to 6 months to evaluate the treatment effect in our clinical practice. Those who were lost at the follow-up or had no record of following lung function were excluded. In total, 95 patients with COPD and 60 patients with asthma were recruited, then followed-up for five years. The study was approved by the Chang Gung Memorial Hospital Ethics Committee (IRB number: 102-4532B) on 18 February 2014.

### 2.2. Clinical Assessment

All subjects performed the 6MWT at baseline. Each participant was instructed to walk back and forth along a 35-meter corridor and stopped walking after 6 min, according to the ATS recommendations [17]. The walking distance was then determined. Oxygen saturation was measured during the procedure with finger pulse oximetry (Criticare Systems Inc., Waukesha, WI, USA). Inspiration capacity (IC) was measured before and after the 6MWT. Pulmonary function tests, including pre-bronchodilator FVC, FEV_1_ and FEV_1_/FVC ratio, were performed using a spirometer every 3 to 6 months (ST-250, Fukuda Sangyo Co. Ltd., Nagareyama, Japan). Desaturation during the 6MWT was defined as a reduction in oxygen saturation (ΔSpO_2_) less than 88%, and the lowest SpO_2_ recorded during the 6MWT was used.

### 2.3. Scoring of High-Resolution Computed Tomography (HRCT)

HRCT (GE SYTEC 3000) was performed to assess the severity and extent of emphysema [26]. Patients were scanned craniocaudally without an infusion of contrast medium in the supine position. The patients held their breath at full inspiration by using 1-millimeter collimation scans (120 kV_p_, 160 mA; scanning time, 3 s). Whole lung images were yielded automatically. Hardcopy images were photographed at a window level of −600 Hounsfield units (HU) and a window width of 1600 HU for appropriate viewing of lung parenchyma. The attenuation coefficient of each pixel was calculated. Mean lung density was defined by the mean attenuation value of all pixels, excluding the mediastinum, trachea and large vessels. Emphysema was defined as areas of hypovascular low attenuation below −950 HU at full inspiration. The emphysema index was measured by calculating the volume fraction of the lungs below −950 HU at full inspiration and the mean lung density at a defined slice. Three HRCT slices (at the level of the carina and 5 cm above and below the carina) of the lung parenchyma were graded for the left and right lung separately, giving six lung fields in total. Severity was graded as follows: 0, no emphysema; 1, low HRCT attenuation areas of <5 mm in diameter with or without vascular pruning; 2, circumscribed low HRCT attenuation areas of >5 mm in diameter; 3, diffuse low attenuation areas. The extent of emphysema using the direct observation method was based on the following four-point scale: 1, 25% of the lung field involved; 2, 25 to 50% involvement; 3, 50 to 75% involvement; and 4, 75 to 100% involvement. The final emphysema score of each lung field was calculated as the severity grade multiplied by the extent, giving a range of scores from 0 to 72. Among the COPD patients, the distinction between predominant emphysema and predominant airway obstruction (no emphysema) on the HRCT was based on a cut-off of 10 of the visual score, according to the previous report [27]. Thus, an HRCT score > 10 was defined as emphysema-predominant COPD (*n* = 62), and an HRCT score ≤ 10 was considered as airway obstruction-predominant COPD (*n* = 33).

### 2.4. Mortality

Survival status and cause of death were verified by using the National Register of Deaths provided by the Taiwan Health and Welfare Data Science Center, Ministry of Health and Welfare (HWDC, MOHW). Causes of death were all-cause mortality (ICD-9 codes: 001–998). Previous studies have validated the accuracy of ICD coding of mortality in the Taiwan deaths registry [28,29].

### 2.5. Statistical Analysis

Descriptive statistics were used to summarize the patient characteristics. Comparisons between groups were performed using the *t*-test for continuous variables and the chi-square test for categorical variables. Overall survival (OS) was defined as the time from the date of enrollment to the date of death or to the last follow-up visit. Cumulative survival curves were performed according to the Kaplan–Meier method and compared with the log-rank test. For pulmonary function that was not measured at regular time-points, a mixed-model repeated-measure analysis was used for subjects with repeated scheduled measurements to examine changes in pulmonary function and to compare the differences between groups. An analysis of covariance was used for a statistical comparison of all-cause mortality between the groups (emphysema-predominant COPD, airway disease-predominant COPD and asthma) adjusted by confounding variables (age; sex; body mass index (BMI); FEV_1_ (%) per 10% decrease; MMEF % predicted; HRCT score; 6MWD per 10 m increase; nadir saturation during 6MWT including oxygen saturation ≥90, 80–89 and <80%); a separate analysis of covariance was performed that was also adjusted by an HRCT score ≤10 or >10 in addition to other confounding variables. The stepwise multivariable regression analysis was used to determine factors of univariate analysis with significant associations with the all-cause mortality adjusted by confounding variables (age; BMI; HRCT score; 6MWD per 10 m increase; Nadir saturation during 6MWT including oxygen saturation ≥90, 80–89 and <80%, or HRCT score ≤10 or >10), then to identify the relative contribution of each variable to predict the five-year mortality. Statistical analyses were performed using SAS 9.2 (SAS Institute, Cary, NC, USA). A *p* value of <0.05 was considered statistically significant.

## 3. Results

### 3.1. Conditions of Asthma and Overall COPD

In total, 60 patients were in the asthma group and 95 were in the COPD group. Overall, 21 patients (35%) from the asthma group and 43 patients (45%) from the COPD group showed desaturation during the 6MWT (*p* = 0.24). The characteristics of these COPD and asthma patients are summarized in Table 1. The COPD group had more males, lower BMIs, higher HRCT scores, lower ICs after the 6MWT, higher ΔICs (post-pre) and lower 6-minute walk distances (6MWD) than the asthma group. There was no difference in age and distribution of lung function severity between the two groups (Table 1). The exertional SpO_2_ during the 6MWT was significantly decreased in the COPD group. Although airflow obstruction was similar between COPD and asthma, the nadir SpO_2_ after the 6MWT was significantly lower in the COPD patients at level of FEV_1_ < 35% of the predicted value (Table 1). There were also no significant differences in the appearance of comorbidities between the three groups.

**Figure 2:**
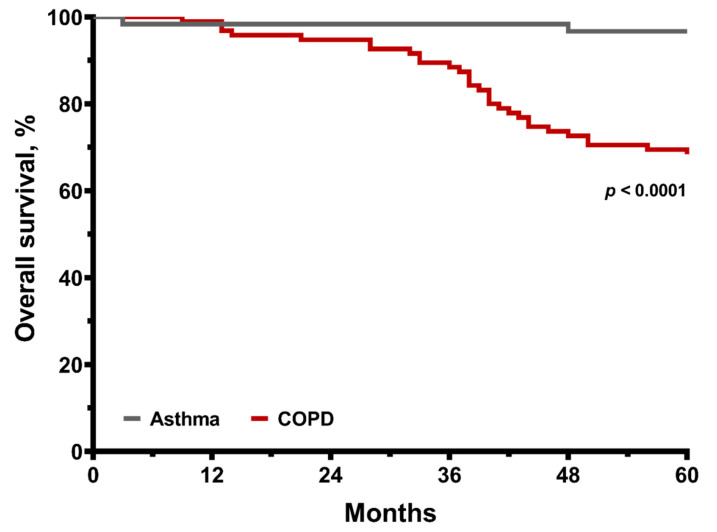
Kaplan–Meier survival curves for matched asthma and COPD cohort for five-year all-cause mortality. The *p* value is shown.

There were 30 (31.6%) deaths among the COPD group and two (3.3%) deaths in the asthma group over the five-year follow-up period (*p* < 0.0001). The Kaplan–Meier plots revealed that the COPD group had a worse overall survival curve than the asthma group over the five-year follow-up period (Figure 2). In univariate analysis, COPD, age, body mass index, body mass index, HRCT score, 6MWD and the lowest SpO_2_ less than 90% during the 6MWT were significantly associated with the overall survival (Table 2), while gender, FEV_1_ and maximal mid-expiratory flow (MMEF%) were not. Then, stepwise multivariate regression analysis (Table 2) was used to evaluate the relative contribution of each variable to predict the five-year mortality. As a result, in the COPD group, the nadir of the lowest SpO_2_ ≤ 80%, HRCT score and age were the selected independent factors for five-year mortality due to a collinearity issue between the variables (Table 2).

### 3.2. Conditions of Asthma and COPD with Predominant Emphysema or Predominant Airway Obstruction

As the emphysema score is a risk factor for COPD mortality, the COPD patients were divided into an emphysema-predominant group (HRCT score > 10, *n* = 62) and an airway obstruction-predominant group (HRCT score ≤ 10, *n* = 33). The emphysema-predominant COPD group had lower BMIs, higher HRCT scores, lower ICs after 6MWT, higher ΔICs (post-pre) and lower 6-minute walk distances (6MWD) than the asthma or the airway obstruction-predominant COPD groups. There was no difference in age and distribution of lung function severity between these three groups (Table 1). The exertional SpO_2_ during the 6MWT was significantly decreased in the emphysema-predominant COPD group. Although airflow obstruction was similar in COPD with or without emphysema and asthma, the nadir SpO_2_ after the 6MWT was significantly lower in the emphysema-predominant COPD patients at a level of FEV_1_ < 35% of the predicted value (Table 1).

**Figure 3:**
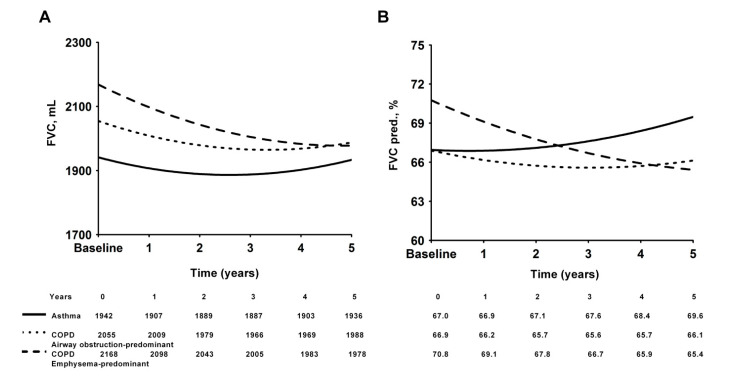
Modeled forced vital capacity (FVC) decline over time in milliliters (**A**) or as a percentage of the predicted value (**B**) in the three groups.

**Figure 4:**
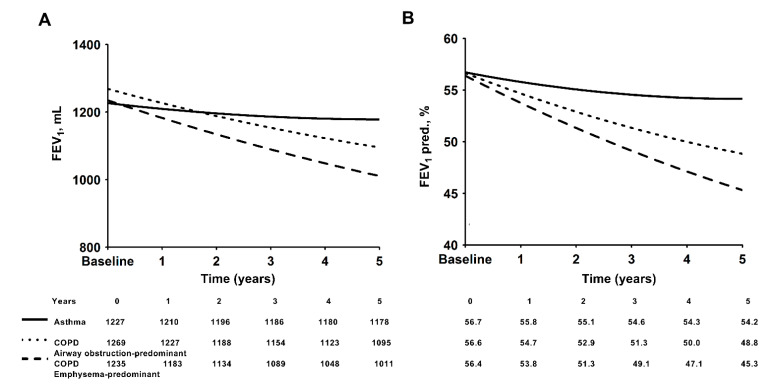
Modeled forced expiratory volume in one second (FEV_1_) decline over time in milliliters (**A**) or as a percentage of the predicted value (**B**) in the three groups.

Using mixed-model repeated-measure models, the estimated declines in FVC and the FVC predicted value (%) as well as the FEV_1_ and the FEV_1_ predicted value (%) are displayed in Figure 3A,B and Figure 4A,B, respectively. The emphysema-predominant COPD group had a significantly greater decline in both FVC and FEV_1_ than the airway obstruction-predominant COPD and the asthma groups. The estimated annual decline in FVC was 38 mL in the emphysema-predominant COPD group, 13.4 mL in the airway disease-predominant COPD group and 1.2 mL in the asthma group (Figure 3A). The estimated annual decline in FEV_1_ was 44.8 mL in the emphysema-predominant COPD group and 34.8 mL in the airway obstruction-predominant COPD group (Figure 4A). The effect of the interaction of time and group (FEV_1_: *p* = 0.037, FVC: *p* = 0.219) and that of time alone (FEV_1_: *p* = 0.003, FVC: *p* = 0.005) were significantly different between the three groups. All regression coefficients for the mixed-model repeated-measure models are shown in the online Supplementary Tables S1–S8.

**Figure 5:**
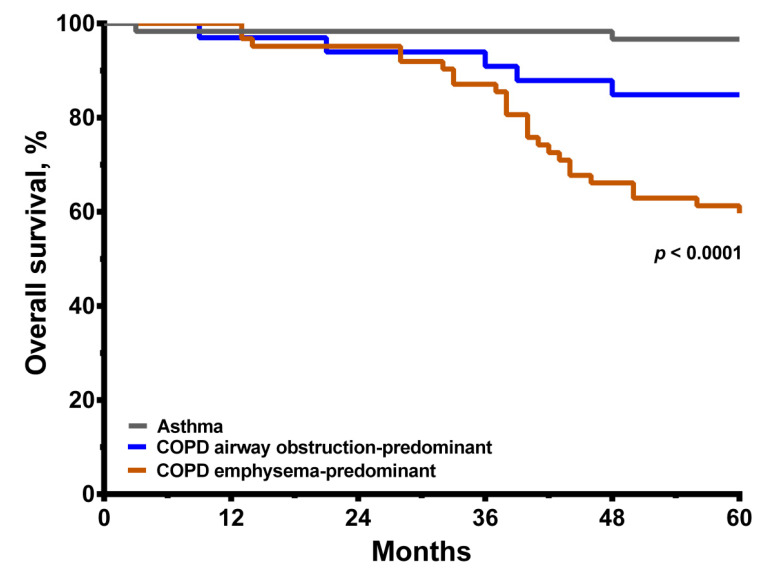
Kaplan–Meier survival curves for matched asthma and COPD with or without emphysema cohort for five-year all-cause mortality. The *p* value is shown.

There were 25 deaths (40.3%) and 5 deaths (15.2%) among the emphysema-predominant COPD and airway obstruction-predominant COPD groups, respectively. The Kaplan–Meier plots revealed that the emphysema-predominant COPD group had a worse overall survival curve than the airway obstruction-predominant COPD group and the asthma group over the five-year follow-up period (*p* < 0.0001, Figure 5). Furthermore, the mortality of the airway obstruction-predominant COPD group was also significantly higher than that of the asthma group (*p* < 0.05). A pulmonary cause was the major cause of death in the emphysema-predominant COPD group. Cerebrovascular disease as a cause of death was also higher in the emphysema-predominant COPD group compared to the airway obstruction-predominant COPD or asthma groups. Other non-respiratory causes of mortality were similar among the three groups. In addition, three of the emphysema-predominant COPD patients died of lung cancer, while none of the patients in the other two groups died of lung cancer, but the statistical analysis did not achieve significant difference. In univariate analysis, COPD with an HRCT score > 10, age, body mass index, HRCT score, 6MWD and the lowest SpO_2_ less than 80% during the 6MWT were significantly associated with the overall survival between the three groups (Table 3), while gender, FEV_1_ and maximal mid-expiratory flow (MMEF%) were not. Then, stepwise multivariate regression analysis (Table 3) was used to evaluate the relative contribution of each variable to predict the five-year mortality. As a result, in the emphysema-predominant COPD group, the nadir of the lowest SpO_2_ ≤ 80%, HRCT score > 10 and age were the selected independent factors for five-year mortality due to a collinearity issue between the variables (Table 3).

## 4. Discussion

We demonstrated that, with a similar degree of airflow obstruction in terms of FEV_1_ and age match, the COPD group had higher emphysema scores associated with a higher Δinspiratory capacity (IC) and lower SpO_2_ during the 6MWT compared to the asthma group. The BMI and 6MWD were significantly lower in the COPD group, which also showed a greater yearly decline in both FEV_1_ (40.6 mL) and FVC (28 mL) than the asthma group (FEV_1_, 9.6 mL; FVC, 1.2 mL) at the five-year follow up. Multivariate analysis revealed that the lowest SpO_2_ ≤ 80% and HRCT score was the strong independent factor for mortality in the COPD group. Thus, we evaluated the impact of emphysema-predominant COPD on the survival rate. We found that the emphysema-predominant COPD group had lower BMIs, higher Δinspiratory capacities (ICs) and lower SpO_2_ during the 6MWT compared to the airway obstruction-predominant COPD group. In the emphysema-predominant COPD group, the lung function had an accelerated yearly decline in FEV_1_ (44.8 mL) and FVC (38 mL) and was associated with a worse survival during the following 5-year period compared to that of the airway obstruction-predominant COPD group. The lowest SpO_2_ ≤ 80% and age were the strong risk factors for mortality in the emphysema-predominant COPD group. Taken together, these data suggest the COPD patients with a greater extent of emphysema and exercise-induced desaturation were prone to an accelerated decline in lung function and worse mortality. 

In the COPD patients, we found that the emphysema score was highly correlated to FVC, FEV_1_ and IC post exercise [30]. Thus, a reduced lung elastic recoil and an expiratory flow limitation led to lung hyperinflation and worse disease progression [31]. In our data, the emphysema-predominant COPD patients had higher emphysema scores associated with a higher Δinspiratory capacity (IC) and lower SpO_2_ during the 6MWT compared to the airway disease-predominant COPD group and the asthma group, even when their initial airflow obstruction was similar. This means that COPD patients had worse lung parenchymal changes than asthma patients, resulting in more dynamic hyperinflation and desaturation during daily activities, even at the similar severity of airflow obstruction. Although the measurement of expiratory flows is a prerequisite for the diagnosis and staging of COPD, the effects of the disease on static and dynamic lung volumes correlate better with patient symptoms and impairment in functional capacity than the spirometric indices of the disease [32]. Though the HRCT revealed no or trivial emphysema in the airway obstruction-predominant COPD group, these patients also had an accelerated annual decline in lung function and worse survival than the asthma group. Based on micro-HRCT imaging analysis, recent studies from James Hogg and colleagues [33,34,35] have shown that narrowing and a reduction in the number of terminal bronchioles appear before, or in parallel with, emphysematous tissue destruction, which could contribute to a rapid decline in FEV₁ or to ventilation–perfusion abnormalities, ultimately leading to severe airway obstruction in COPD. Hyperinflation is clinically relevant for patients with COPD, mainly because it contributes to the morbidity associated with the disease progression [36].

The annual decline in FEV_1_ is generally used as a marker of disease progression. A review of large longitudinal studies found that the mean rate of FEV_1_ decline was highest at Global Initiative for Chronic Obstructive Lung Disease (GOLD) stage II (47–79 mL·year^−1^), indicating the need for intervention at earlier stages of the disease [37]. Fletcher et al. [38] also reported that smokers with airway obstruction had a rapid annual decline in FEV_1_ of 60–80 mL per year, but that this could be slowed by stopping smoking. In addition, Vestbo et al. [39] reported that COPD patients who were current smokers or had emphysema had an excessive loss of FEV_1_ compared to those who were ex-smokers or did not have emphysema. Furthermore, Cerveri et al. [40] noted that the presence of emphysema in a COPD patient was an independent predictor of a rapid decline in FEV_1_, and that an increased residual volume, the physiological hallmark of emphysema, was also associated with a rapid decline in FEV_1_ [40]. COPD associated with more severe emphysema on HRCT is characterized by more severe lung function impairment, greater exercise impairment and cardiopulmonary dysfunction [30]. FEV_1_ is influenced by both airway resistance and reduced elastic recoil caused by emphysema, hence the association between the baseline radiological burden of emphysema and the subsequent decline in FEV_1_ [39,41]. In our results, the COPD group had higher emphysema scores and a greater yearly decline in both FEV_1_ (40.6 mL) and FVC (28 mL) than the asthma group (FEV_1_, 9.6 mL; FVC, 1.2 mL; *p* < 0.05), which is consistent with the rapid FEV_1_ decline in COPD being associated with predominant emphysema [39,40]. This means that the lesser parenchymal change in asthma is the reason for little or lesser yearly decline in lung function. Airway inflammation is a major pathophysiological problem in asthma. When it is controlled adequately by regular inhaled corticosteroid therapy or combined with a long-acting beta agonist, lung function remains stable and mortality has been shown to be reduced [42,43]. In contrast, even under treatment with dual bronchodilators or other anti-inflammatory agents, lung function still declines progressively in COPD. Other factors apart from airway inflammation contribute to the higher mortality of chronic obstructive airway disease.

COPD patients with advanced emphysema have significant mortality. Subgroups based on age, oxygen utilization, physiological measures, exercise capacity and emphysema distribution contribute to an increased risk of death [44]. Exercise-induced oxygen desaturation occurs in about 20% of COPD patients. The underlying physiological determinants include the presence of advanced emphysema, a severe airflow limitation and low oxygen saturation at rest [45]. Oxygen desaturation during the 6MWT in COPD patients is an important predictor of mortality, exacerbations, decline in lung function and loss of lean body mass [19]. In patients with COPD, desaturation during exertion has been shown to primarily be a consequence of an insufficient increase in ventilation due to hyperinflation and possibly insufficient cardiac output or increased peripheral oxygen extraction [46,47]. However, it could be that exertional desaturation in itself is harmful. Therefore, on-and-off desaturation occurring when the patient is exercising could lead to similar long-term effects, such as persistent hypoxemia, due to a possible repeated micro-trauma caused by desaturation [31]. Another possibility is that the accompanying hypoxia and hypoxaemia may promote bacterial infection [48], enhance the activation of hypoxia-inducible factors (HIF) and nuclear factor (NF)-κB [49] and propagate systemic inflammation or increase recurrent exacerbations [49,50]; this would contribute to the development of adverse sequelae in COPD patients, such as pulmonary hypertension, secondary polycythemia, skeletal muscle dysfunction, systemic inflammation and neurocognitive dysfunction [47]. These will ultimately influence the overall mortality of COPD patients. However, exertional dyspnea occurred less often in the asthmatic patients due to less or no parenchymal destruction. Therefore, in our study, we concluded that the emphysema-predominant COPD patients had greater desaturation during the 6MWT (SpO_2_ less than 80%), which led to greater mortality than that of the asthma patients or the airway obstruction-predominant COPD group with a similar airflow obstruction.

### Limitations

There are some limitations to this study. First, a prospective study to evaluate the long-term outcome of COPD and asthma overlap syndrome and asthma alone would consolidate our evidence. Secondly, we did not measure 24 h SpO_2_ to ascertain whether the non-survival of COPD patients would decrease the time of desaturation during daily activities. Thirdly, the progression of COPD is related to the clinical phenotype; thus, classifying COPD patients by their clinical phenotypes can identify clusters of subjects with different mortality outcomes. Fourthly, the diffusing capacity of the lung for carbon monoxide (DLCO) has been reported to be an independent factor for mortality in stable COPD patients [50,51]. Our study observed whether the exercise-induced desaturation was associated with mortality in COPD and asthma patients. Therefore, we did not measure the DLCO. We may conduct a study to clarify whether the DCLO, emphysema score and the lowest SpO_2_ during the 6MWT have more impact on the mortality of COPD patients in the future.

## 5. Conclusions

In conclusion, a higher emphysema score and a greater desaturation during the 6MWT in COPD may be associated with a worse yearly decline of FEV_1_ and higher five-year mortality rate than those in asthma patients with a similar airflow obstruction. COPD with an HRCT score > 10 and the lowest SpO_2_ < 80% during the 6MWT are independently associated with 5-year mortality in patients with COPD and asthma. An early alert of these two risk factors may raise clinician awareness to promote early intervention, thereby preventing the development of adverse sequelae and increased mortality in COPD patients.

## Figures and Tables

**Table 1:** Baseline characteristics of asthma and COPD with predominant emphysema or predominant airway obstruction.

	COPD Total (N = 95)	COPD Emphysema- Predominant (N = 62)	COPD Airway Obstruction- Predominant (N = 33)	Asthma (N = 60)	*p*-Value
Age, years	67.1 ± 9.1	67.1 ± 9.0	67.3 ± 9.3	64.8 ± 12	0.387
Gender, female	8	2	5	20	<0.001
Body mass index, kg/m^2^	23.0 ± 3.5	22.6 ± 3.4	23.7 ± 3.8	25.3 ± 3.1	<0.001
Smoking habit	84	56	28	6	<0.001
HRCT score	22.3 ± 19.6	30.8 ± 19.5	6.4 ± 1.7	1.8 ± 3.2	<0.001
Appearance of comorbidities Ischemic heart disease					0.992
Ischemic heart disease	14	8	6	9	0.788
Cerebrovascular disease	4	2	2	2	0.803
Diabetes	13	9	4	11	0.704
Liver disease	2	1	1	2	0.822
Chronic kidney disease	7	5	2	5	0.948
Pulmonary function (pre-bronchodilator)					
FVC, L	2.13± 0.54	2.15± 0.55	2.10 ± 0.51	2.00± 0.50	0.315
FVC, % pred.	69.61 ± 17.45	69.75 ± 18.58	69.34± 15.36	72.70± 13.82	0.510
FEV_1_, L	1.25 ± 0.44	1.23 ± 0.41	1.28 ± 0.48	1.20 ± 0.38	0.672
FEV_1_, % pred.	56.70 ± 21.89	55.90 ± 22.16	58.22 ± 21.63	59.04 ± 19.66	0.702
FEV_1_/FVC, %	58.10 ± 11.99	57.19 ± 11.29	59.8 2± 13.21	60.61± 14.06	0.318
FEV_1_: <35%	15	9	6	7	0.769
FEV_1_: 35~50%	20	16	4	15	
FEV_1_: 50~75%	44	28	16	28	
FEV_1_: >75%	16	9	7	10	
MMEF, % pred.	25.23 ± 14.43	25.10± 14.84	25.48 ± 13.84	25.29 ± 14.98	0.994
Pre-6MWT IC, L	1.50 ± 0.45	1.51 ± 0.47	1.48± 0.43	1.57 ± 0.36	0.588
Post-6MWT IC, L	1.39 ± 0.43 *	1.34± 0.40	1.49± 0.48	1.57 ± 0.42	0.029
ΔIC (post-pre)	−0.11 ± 0.27 *	-0.18 ± 0.19	0.01 ± 0.34	0.00 ± 0.25	0.001
6MWD, M	387.7 ± 101.0 *	380.4 ± 107.2	404.4 ± 87.3	432.0 ± 111.9	0.028
SpO_2_ saturation					
Pre-exercise, %	95.2 ± 2.1	95.0 ± 2.3	95.3 ± 1.6	95.5 ± 2.3	0.535
Post-exercise, %	87.1 ± 7.0 **	86.4 ± 6.7	88.3 ± 7.4	89.7 ± 3.9	0.006
Post, SpO_2_					
FEV_1_: <35%	84.0 (66, 91) **	83.0 (66, 91)	86.5 (80, 90)	89.0 (85, 91)	0.016
FEV_1_: 35~50%	88.0 (74, 95)	87.0 (74, 93)	91.0 (79, 95)	90.0 (77, 95)	0.156
FEV_1_: 50~75%	90.5 (71, 97)	90.0 (71, 95)	91.0 (72, 97)	90.5 (81, 98)	0.801
FEV_1_: >75%	91.5 (59, 93)	89.0 (72, 93)	93.0 (59, 93)	91.5 (84, 94)	0.459
Overall mortality event					0.0001
All-cause death, *n* (%)	30 (31.6)	25 (40.3)	5 (15.2)	2 (3.3)	<0.0001
Cardiovascular death, *n* (%)	4 (4.2)	4 (6.5)	0 (0.0)	0 (0.0)	0.046
Lung cancer death, *n* (%)	3 (3.2)	3 (4.8)	0 (0.0)	0 (0.0)	0.101
Pulmonary cause, *n* (%)	17 (17.9) **	14 (22.6)	3 (9.1)	0 (0.0)	0.0003
Other death, *n* (%)	6 (6.3)	4 (6.4)	2 (6.1)	2 (3.3)	0.714
Disease-specific death (pulmonary, cardiovascular and lung cancer), *n* (%)	24 (25.3) **	21 (33.9)	3 (9.1)	0 (0.0)	<0.0001

Note: Data are presented as mean ± standard deviation or number (percentage). Abbreviations: COPD, chronic obstructive pulmonary disease; HRCT, high-resolution computed tomography; FVC, forced volume capacity; FEV_1_, forced expiratory volume in one second; MMEF, maximal mid-expiratory flow; IC, inspiratory capacity; 6MWT, six-minute walk test; SpO_2_, oxygen saturation by pulse oximetry; 6MWD, six-minute walk distance. * *p* < 0.05, ** *p* < 0.01 compared to the group of asthma. The *p*-value indicated the statistical difference between the three groups (emphysema-predominant COPD, airway obstruction-predominant COPD and asthma).

**Table 2:** Factors associated with all-cause mortality at a five-year follow-up in a Cox regression model.

	Univariate			Multivariate		
	HR	95% CI	*p*-Value	HR	95% CI	*p*-Value
Groups						
Asthma	1 (ref)			1 (ref)		
COPD	11.036	2.636–46.203	0.001	6.979	0.852–57.173	0.070
Age, years	1.055	1.013–1.099	0.011	1.055	1.000–1.113	0.048
Gender						
Female	1 (ref)					
Male	7.199	0.983–52.741	0.052			
Body mass index	0.885	0.796–0.983	0.023	1.016	0.906–1.139	0.791
FEV_1_ (%) per 10% decrease	1.000	0.843–1.185	0.999			
MMEF % pred. value	0.998	0.972–1.025	0.880			
HRCT score	1.031	1.016–1.046	<0.001	1.021	1.000–1.043	0.048
6MWD per 10 m increase	0.954	0.926–0.983	0.002	0.994	0.957–1.033	0.767
Nadir saturation during 6MWT						
SpO_2_: ≥90%	1 (ref)			1 (ref)		
SpO_2_: 80–89%	2.738	1.616–6.461	0.021	2.120	0.761–5.902	0.150
SpO_2_: <80%	7.019	2.669–18.252	<0.001	4.918	1.556–15.550	0.007

Abbreviations: COPD, chronic obstructive pulmonary disease; FEV_1_, forced expiratory volume in 1 s; MMEF, maximal mid-expiratory flow; HRCT, high-resolution computed tomography; 6MWD, six-minute walk distance; 6MWT, six-minute walk test; SpO_2_, oxygen saturation by pulse oximetry; HR: hazard ratio; CI: confidence interval.

**Table 3:** Factors associated with all-cause mortality at a five-year follow-up in a Cox regression model.

	Univariate			Multivariate		
	HR	95% CI	*p*-Value	HR	95% CI	*p*-Value
Groups						
Asthma	1 (ref)			1 (ref)		
COPD HRCT score ≤ 10	4.929	0.956–25.407	0.057	4.947	0.531–46.134	0.160
COPD HRCT score > 10	14.726	3.485–26.229	<0.001	9.817	1.110–86.785	0.040
Age, years	1.055	1.013–1.099	0.011	1.056	1.001–1.115	0.046
Gender						
Female	1 (ref)					
Male	7.199	0.983–52.741	0.052			
Body mass index	0.885	0.796–0.983	0.023	1.009	0.895–1.136	0.887
FEV_1_ (%) per 10% decrease	1.000	0.843–1.185	0.999			
MMEF % pred. value	0.998	0.972–1.025	0.880			
HRCT score	1.031	1.016–1.046	<0.001	1.014	0.989–1.040	0.283
6MWD per 10 m increase	0.954	0.926–0.983	0.002	0.999	0.961–1.039	0.966
Nadir saturation during 6MWT						
SpO_2_: ≥90%	1 (ref)			1 (ref)		
SpO_2_: 80–89%	2.738	1.161–6.461	0.021	2.403	0.736–5.675	0.170
SpO_2_: <80%	7.019	2.699–18.252	<0.001	4.859	1.537–15.353	0.007

Abbreviations: COPD, chronic obstructive pulmonary disease; FEV_1_, forced expiratory volume in 1 s; MMEF, maximal mid-expiratory flow; HRCT, high-resolution computed tomography; 6MWD, six-minute walk distance; 6MWT, six-minute walk test; SpO_2_, oxygen saturation by pulse oximetry; HR, hazard ratio; CI, confidence interval.

## Data Availability

The data will not be shared according to the regulations of Chang Gung Memorial Hospital IRB for patient confidentiality.

## Supplementary Materials

The following are available online at https://www.mdpi.com/article/10.3390/medicina57111261/s1: Table S1. All regression coefficients for the mixed-model repeated-measure model for FEV_1_ (forced expiratory volume in one second, mL). Table S2. All regression coefficients for the mixed-model repeated-measure model for FEV_1_ predicted value (%). Table S3. All regression coefficients for the mixed-model repeated-measure model for FVC (forced vital capacity, mL). Table S4. All regression coefficients for the mixed-model repeated-measure model for FVC predicted value (%). Table S5. All regression coefficients for the mixed-model repeated-measure model for FEV_1_ (forced expiratory volume in one second, mL) in three groups. Table S6. All regression coefficients for the mixed-model repeated-measure model for FEV_1_ predicted value (%) in the three groups. Table S7. All regression coefficients for the mixed-model repeated-measure model for FVC (forced vital capacity, mL) in three groups. Table S8. All regression coefficients for the mixed-model repeated-measure model for FVC predicted value (%) in the three groups.
