# Supplementary material for: Emphysema-Predominant COPD Had a Greater 5-Year Mortality and a Worse Annual Decline in Lung Function Than Airway Obstruction-Predominant COPD or Asthma at Initial Same Degree of Airflow Obstruction
Source: Medicina (Kaunas). 2021 Nov 17;57(11):1261. doi: 10.3390/medicina57111261 (PMC8622286; doi:10.3390/medicina57111261)
Supplement: Supplementary file 1 [file medicina-57-01261-s001.zip › medicina-1409425-Supplementary.pdf]

Online supplementary tables

Table S1. All regression coefficients for the mixed-model repeated-measure model for FEV_1 (forced expiratory volume in one second, mL).

Solution for Fixed Effects						
Effect	group	Estimate	Standard Error	DF	t Value	*p*-value
Intercept		1246.420	44.995	152	27.70	<.0001
time		-4.202	1.114	143	-3.77	0.0002
group	Asthma	-19.670	71.874	938	-0.27	0.7844
group	COPD	0	.	.	.	.
time*group	Asthma	2.597	1.041	938	2.50	0.0127
time*group	COPD	0	.	.	.	.
time*time		0.013	0.020	938	0.69	0.4916

Type 3 Tests of Fixed Effects				
Effect	Num DF	Den DF	F Value	*p*-value
time	1	143	7.74	0.0061
group	1	938	0.07	0.7844
time*group	1	938	6.23	0.0127
time*time	1	938	0.47	0.4916

Table S2. All regression coefficients for the mixed-model repeated-measure model for FEV_1 predicted value (%).

Solution for Fixed Effects						
Effect	group	Estimate	Standard Error	DF	*t* Value	*p*-value
Intercept		56.469	2.074	152	27.22	<.0001
time		-0.209	0.060	143	-3.49	0.0007
group	Asthma	0.261	3.301	938	0.08	0.9371
group	COPD	0	.	.	.	.
time*group	Asthma	0.121	0.058	938	2.07	0.0389
time*group	COPD	0	.	.	.	.
time*time		0.001	0.001	938	0.74	0.4614

Type 3 Tests of Fixed Effects				
Effect	Num DF	Den DF	F Value	*p*-value
time	1	143	7.06	0.0088
group	1	938	0.01	0.9371
time*group	1	938	4.28	0.0389
time*time	1	938	0.54	0.4614

Table S3. All regression coefficients for the mixed-model repeated-measure model for FVC (forced vital capacity, mL).

Solution for Fixed Effects						
Effect	group	Estimate	Standard Error	DF	*t* value	*p*-value
Intercept		2128.370	55.582	152	38.29	<.0001
time		-5.858	1.793	143	-3.27	0.0014
group	Asthma	-186.540	87.958	938	-2.12	0.0342
group	COPD	0	.	.	.	.
time*group	Asthma	2.238	1.585	938	1.41	0.1582
time*group	COPD	0	.	.	.	.
time*time		0.059	0.032	938	1.83	0.0677

Type 3 Tests of Fixed Effects				
Effect	Num DF	Den DF	F Value	*p*-value
time	1	143	7.89	0.0057
group	1	938	4.50	
time*group	1	938	1.99	0.1582
time*time	1	938	3.35	0.0677

Table S4. All regression coefficients for the mixed-model repeated-measure model for FVC predicted value (%).

Solution for Fixed Effects						
Effect	group	Estimate	Standard Error	DF	*t* value	*p*-value
Intercept		69.400	1.678	151	41.36	<.0001
time		-0.122	0.074	142	-1.65	0.1002
group	Asthma	-2.430	2.622	936	-0.93	0.3543
group	COPD	0	.	.	.	.
time*group	Asthma	0.101	0.068	936	1.49	0.1354
time*group	COPD	0	.	.	.	.
time*time		0.001059	0.001301	936	0.81	0.4162

Type 3 Tests of Fixed Effects				
Effect	Num DF	Den DF	F Value	*p*-value
time	1	142	1.06	0.3053
group	1	936	0.86	0.3543
time*group	1	936	2.23	0.1354
time*time	1	936	0.66	0.4162

Table S5 All regression coefficients for the mixed-model repeated-measure model for FEV_1 (forced expiratory volume in one second, mL) in three groups.

Solution for Fixed Effects						
Effect	group	Estimate	Standard Error	DF	t Value	*p*-Value
Intercept		1226.550	56.576	150	21.68	<.0001
time		-1.568	1.214	140	-1.29	0.1986
group	COPD HRCT score≦10	-10.780	85.039	936	-0.13	0.8992
group	COPD HRCT score >10	41.215	85.030	936	0.48	0.6280
group	Asthma	0	.	.	.	.
time*group	COPD HRCT score≦10	-1.702	1.214	936	-1.40	0.1615
time*group	COPD HRCT score >10	-3.635	1.217	936	-2.99	0.0029
time*group	Asthma	0	.	.	.	.
time*time		0.013	0.020	936	0.64	0.5218

Type 3 Tests of Fixed Effects				
Effect	Num DF	Den DF	F Value	*p*-Value
time	1	140	10.32	0.0016
group	2	936	0.19	0.8279
time*group	2	936	4.47	0.0117
time*time	1	936	0.41	0.5218

Table S6 All regression coefficients for the mixed-model repeated-measure model for FEV1 predicted value (%) in the three groups.

**Solution for Fixed Effects**						
Effect	group	Estimate	Standard Error	DF	t Value	*p*-Value
Intercept		56.718	2.603	151	21.79	<.0001
time		-0.086	0.066	141	-1.30	0.1951
group	COPD HRCT score ≦10	-0.074	4.352	939	-0.02	0.9865
group	COPD HRCT score >10	-0.332	3.640	939	-0.09	0.9273
group	Asthma	0	.	.	.	.
time*group	COPD HRCT score ≦10	-0.087	0.074	939	-1.18	0.2396
time*group	COPD HRCT score >10	-0.142	0.065	939	-2.18	0.0296
time*group	Asthma	0	.	.	.	.
time*time		0.001	0.001	939	0.69	0.4905

**Type 3 Tests of Fixed Effects**				
Effect	Num DF	Den DF	F Value	P-Value
time	1	141	8.32	0.0045
group	2	939	0.00	0.9956
time*group	2	939	2.41	0.0908
time*time	1	939	0.48	0.4905

Table S7 All regression coefficients for the mixed-model repeated-measure model for FVC (forced vital capacity, mL) in three groups.

Solution for Fixed Effects						
Effect	group	Estimate	Standard Error	DF	t Value	*P*-Value
		1940.730	68.543	150	28.31	<.0001
time	Intercept	-3.441	1.929	140	-1.78	0.0767
group	COPD HRCT score ≦10	55.798	103.090	936	0.54	0.5884
group	COPD HRCT score >10	313.680	103.090	936	3.04	0.0024
group	Asthma	0	.	.	.	.
time*group	COPD HRCT score ≦10	-0.894	1.850	936	-0.48	0.6290
time*group	COPD HRCT score >10	-3.872	1.859	936	-2.08	0.0376
time*group	Asthma	0	.	.	.	.
time*time		0.055	0.032	936	1.71	0.0882

Type 3 Tests of Fixed Effects				
Effect	Num DF	Den DF	F Value	*P*-Value
time	1	140	8.89	0.0034
group	2	936	5.01	0.0069
time*group	2	936	2.27	0.1039
time*time	1	936	2.91	0.0882

Table S8. All regression coefficients for the mixed-model repeated-measure model for FVC predicted value (%) in the three groups.

Solution for Fixed Effects						
Effect	group	Estimate	Standard Error	DF	t Value	*p*-Value
Intercept		66.949	2.073	150	32.30	<.0001
time		-0.017	0.080	141	-0.21	0.8314
group	COPD HRCT score ≦10	-0.049	3.450	936	-0.01	0.9886
group	COPD HRCT score >10	3.805	2.887	936	1.32	0.1879
group	Asthma	0	.	.	.	.
time*group	COPD HRCT score ≦10	-0.0551	0.086	936	-0.64	0.5212
time*group	COPD HRCT score >10	-0.1311	0.076	936	-1.73	0.0832
time*group	Asthma	0	.	.	.	.
time*time		0.00099	0.001304	936	0.76	0.4478

Type 3 Tests of Fixed Effects				
Effect	Num DF	Den DF	F Value	P-Value
time	1	141	1.30	0.2569
group	2	936	1.06	0.3464
time*group	2	936	1.51	0.2213
time*time	1	936	0.58	0.4478
